# Postpartum needs and experiences of young and first-time mothers in northwestern Tanzania

**DOI:** 10.3389/ijph.2026.1609545

**Published:** 2026-10-07

**Authors:** Esther Y. Shija, Janeth Peter, Elias C. Nyanza, Namanya Basinda, Joseph R. Mwanga

**Affiliations:** 1 Department of Epidemiology, Biostatistics and Behavioral Sciences, School of Public Health, Catholic University of Health and Allied Sciences, Mwanza, Tanzania; 2 Department of Community Medicine and Environmental Health, School of Public Health, Catholic University of Health and Allied Sciences, Mwanza, Tanzania

**Keywords:** first-time mothers, lived experiences, postnatal care, Postpartum Needs, postpartum period

## Abstract

**Objective:**

To explore postpartum needs and lived experiences of young and first-time mothers in northwestern Tanzania.

**Methods:**

This descriptive, phenomenological, qualitative study was conducted between April and July 2022. Participants included young mothers (<20 years old) and first-time mothers within 6 weeks postpartum. Data were collected via 20 in-depth interviews (IDIs) and seven focus group discussions (FGDs) with 42 participants from government health facilities in Mwanza City. The interviews were audio-recorded, transcribed, translated into English, and analyzed using inductive thematic analysis.

**Results:**

The majority of participants (77.8%) were first-time mothers; 69.4% of the respondents were aged 25–35 years. Three themes emerged: postpartum needs, positive experiences, and negative experiences. Postpartum needs included informational support, psychological support, and practical assistance. Positive experiences included pride in motherhood, bonding with the infant, and increased confidence. Negative experiences included sleep disturbances, breastfeeding challenges, changes in appetite, anxiety, and emotional distress, exacerbated by limited support.

**Conclusion:**

Young and first-time mothers face significant physical, emotional, and informational challenges in the postpartum period. Strengthening social support and targeted postnatal interventions is critical to improving maternal and infant outcomes in high-burden settings.

## Introduction

The transition to motherhood is a significant life event that requires emotional, psychological, and social adjustments, particularly for young, first-time mothers who often have limited parenting experience and support [1, 2]. The postpartum period (the first 6 weeks after childbirth) is critical for recovery, yet it is frequently overlooked [3]. Globally, a large proportion of maternal and neonatal deaths occur during this time, with sub-Saharan Africa shouldering the heaviest burden [4]. In Tanzania, despite progress in reproductive, maternal, newborn, child, and adolescent health (RMNCAH) policies, postpartum care—especially psychosocial support—remains insufficient.

Young mothers (<20 years old) and first-time mothers are especially vulnerable due to inadequate preparation, fragmented information from family/peers, and limited healthcare guidance [4]. Studies from Uganda, Kenya, and elsewhere in sub-Saharan Africa highlight common challenges, including breastfeeding difficulties, anxiety, stigma, and insufficient emotional/practical support [5]. In Tanzania, harmful practices and low utilization of postnatal services further elevate the risks for younger mothers [5]. In Uganda, teenage first-time mothers reported significant healthcare needs in the immediate and early postpartum period, including information on self-care, newborn care, and emotional support; many of them felt unprepared, experienced anxiety related to breastfeeding and infant crying, faced stigma, or had limited family support [6]. Another phenomenological study of adolescent mothers at Mbale Regional Referral Hospital, Uganda, highlighted hardships such as fear of the unknown during the intrapartum and immediate postpartum periods, negative treatment by family and community members, extreme parenting challenges, and social isolation—although the joy of seeing their babies often alleviated some of the stress [7].

Qualitative findings from Kenya further illustrate these issues. First-time mothers in Nairobi reported breastfeeding difficulties in the early postpartum period, influenced by inadequate information, pain, and a lack of practical support from healthcare workers or family [8]. Broader reviews of postnatal care experiences in sub-Saharan Africa consistently reveal inadequate information provision, poor communication with healthcare workers, limited resources, and insufficient emotional support, which exacerbate feelings of isolation and anxiety among young and first-time mothers [9].

Globally, adolescent pregnancy remains a public health concern. The World Health Organization (WHO) estimates that approximately 21 million girls aged 15–19 years in developing countries become pregnant each year, with approximately 12 million of them giving birth [10]. This group is at increased risk of pregnancy-related complications, adverse birth outcomes, and poor utilization of maternal health services [11–13].

In Tanzania, substantial efforts have been made to improve reproductive, maternal, newborn, child, and adolescent health (RMNCAH) through national policies and initiatives, including the Sharpened One Plan and the Health Sector Strategic Plan IV [14]. Despite these efforts, postpartum care—particularly psychosocial support—remains insufficient. Evidence suggests that first-time mothers require emotional, instrumental, and informational support to effectively navigate the postpartum period [15]. However, such support is often inadequate in low-resource settings [15–17].

The Mwanza Region in northwestern Tanzania has a high rate of institutional maternal deaths. According to the national Maternal and Perinatal Death Surveillance and Response (MPDSR) Report for 2018–2023, Mwanza Region recorded 962 institutional maternal deaths (the second highest total nationally after Dar es Salaam), with Mwanza City Council ranking second among city councils with 511 deaths (5.3% of the national total) [18]. This is linked to suboptimal coverage of antenatal care (≥4 visits), skilled birth attendance, and postnatal care, alongside prevalent conditions such as maternal anemia, malaria during pregnancy, and HIV [18].

This study aimed to explore the postpartum needs and lived experiences of young and first-time mothers in Mwanza City to inform context-specific interventions that would enhance maternal and infant health in high-mortality urban settings.

## Methods

### Study design

This study employed a descriptive, phenomenological, qualitative design grounded in Husserlian phenomenology. This approach seeks to describe the essence of lived experiences as perceived by participants, while researchers bracket (epoché) preconceptions to minimize bias. This design is well-suited to exploring subjective meanings of the postpartum period among young and first-time mothers [18].

### Study population

The study population consisted of women of reproductive age (15–49 years) who were either first-time mothers (regardless of age) or young mothers under 20 years old (regardless of parity).

#### Inclusion criteria

- Women who had given birth within the past 6 weeks (to minimize recall bias).
- Able to speak Kiswahili fluently.
- Willing and able to provide informed consent (or assent for those under 18 years old, with parental/guardian consent where required).
- Attending postnatal care or child health services at the selected government health facilities.

#### Exclusion criteria

- Women who had experienced severe obstetric complications (e.g., stillbirth, neonatal death, or major postpartum hemorrhage requiring intensive care), as these could significantly alter their postpartum experience.
- Women who were too ill to participate in an interview or discussion.
- Non-residents of Mwanza City who were only visiting for care.

### Study setting

The study was conducted in Mwanza City (Nyamagana and Ilemela districts), northwestern Tanzania. According to the 2022 National Population and Housing Census, Mwanza City had a population of approximately 1.22 million. The study was health facility–based and conducted in six wards (three from each district) using government health facilities that provide maternal and child health services.

The Mwanza Region was purposively selected due to its high burden of institutional maternal mortality. From 2018 to 2023, it recorded 962 institutional maternal deaths, while Mwanza City Council recorded 511 deaths, the second-highest among city councils nationally [18].

This burden is associated with gaps in antenatal, skilled delivery, and postnatal care, alongside conditions such as maternal anemia, malaria, and HIV-related complications [19, 20]. These challenges make Mwanza City an important setting for studying postpartum experiences among young and first-time mothers.

### Sample size and sampling procedure

A total of 62 participants were recruited: 20 IDIs and 7 FGDs (6 participants each, n = 42). Purposive sampling with maximum variation (in terms of age, parity for young mothers, education, marital status, and residence) was used. Recruitment occurred post-clinic visits via trained research assistants. IDI participants were excluded from FGDs to prevent bias. Sampling continued until data saturation (i.e., no new themes emerged), confirmed after 15 IDIs and 6 FGDs, with additional data collection conducted for robustness.

Wards were purposively selected based on 2022 census data and categorized as either high- or low-population density areas to capture socio-economic diversity. Within these wards, government health facilities offering maternal and child health services were purposively chosen. Eligible participants were identified from maternal health registers (from postnatal and child welfare clinics).

Recruitment followed a purposive sampling strategy to achieve maximum variation in age, parity (for young mothers), education level, marital status, and place of residence. Potential participants were approached face-to-face by trained research assistants after their routine clinic visits. They received detailed study information and provided with written informed consent (or assent for those under 18 years old). Participants selected for IDIs were excluded from FGDs to avoid response bias and contamination of group dynamics.

### Data collection

Data collection involved triangulation of in-depth interviews and focus group discussions. Four trained qualitative researchers conducted all interviews using pre-tested semi-structured guides. The interviews were conducted in Kiswahili, audio-recorded, and supplemented with field notes. IDIs lasted 30–45 min, while FGDs lasted 1–2 h. Socio-demographic data were also collected.

### Data analysis

The interviews were transcribed verbatim, translated into English, and verified against the recordings. Inductive thematic analysis was performed using NVivo version 15. Two investigators independently familiarized themselves with the data, developed a coding framework, coded independently, and resolved discrepancies through discussion. The codes were grouped into categories, sub-themes, and major themes. Findings were supported by illustrative participant quotations.

The two investigators independently applied the agreed coding framework to the transcripts, after which the codes were compared and refined. The researchers discussed similarities and differences and organized the codes into broader categories, themes, and sub-themes. Any discrepancies in the coding were resolved through discussion and consensus to ensure consistency and rigor in the analysis. The final themes were refined and clearly defined, and the findings were presented in narrative form, supported by illustrative quotations from participants.

### Ethical considerations

Ethical approval was obtained from the Joint Catholic University of Health and Allied Sciences/Bugando Medical Centre Research Ethics Committee (CREC/539/2022). Written informed consent/assent was obtained, and confidentiality/voluntary participation was maintained.

## Results

### Sociodemographic characteristics of the study participants

Table 1 presents the sociodemographic characteristics of the study participants. A total of 62 young and first-time mothers participated, including 20 taking part in in-depth interviews and 42 in focus group discussions. The majority of participants were first-time mothers (77.8%), while 24.2% were young mothers. Educational attainment varied, with 30.6% having received a primary education, 41.9% a secondary education, and 27.4% a higher education. The majority of participants were aged 25–35 years (69.4%), followed by those aged 15–25 years (17.7%).

**TABLE 1:** Sociodemographic characteristics of the study participants (Mwanza region, Tanzania,2022).

Variables	Category	Number (N)	Percentage (%)
Mothers	First-time mothers	47	77.8
	Young mothers (<20 years old)	15	24.2
Age (years)	15–25	11	17.7
	25–35	43	69.4
	35–45	8	12.9
Marital status	Married	50	80.6
	Unmarried	12	19.4
Education	Primary	19	30.6
	Secondary	26	41.9
	Higher education	17	27.4

### Post-partum needs of young and first-time mothers

The findings revealed several key themes regarding the postpartum needs of young and first-time mothers, including informational needs, psychological support, and practical and material support in both home and hospital settings (see Table 2).

**TABLE 2:** Needs of first-time mothers during the postpartum period (Mwanza region, Tanzania,2022).

Theme	Sub-theme	Description
Postpartum needs	Informational needs	Lack of adequate information on motherhood, newborn care, and postpartum challenges
	Psychological support	Need for emotional support, reassurance, and someone to share experiences with
	Practical support	Need for assistance with childcare, household activities, and rest
	Material/Financial support	Need for basic items (e.g., clothes, soap) and financial preparedness

### Informational needs

Participants reported insufficient preparation for motherhood and expressed a strong need for early and continuous education. In one of the FGDs, a mother mentioned,

“*Even so, during pregnancy, we are in something else, and we’re probably not ready to hear it, we would have liked to be prepared early, and be informed on what will happen after (first baby)” (FGD032022, Nyamagana*).

Another *mother* added*, “I would have liked it if someone had talked to me before about infant acne, taking care of the cord, and about the weird sounds that a baby makes because sometimes it's scary” (IDI022022, Nyamagana).*

### Need for psychological support

Many participants described feelings of isolation and emotional distress, highlighting the importance of social and emotional support. One mother, during an in-depth interview, pointed out that

“A mother should have a reference person during pregnancy, such as a friend, relative, or nurse, who can provide support and share experiences.” (IDI932022R1Hosp).

Another participant added, *“I have my best friend, who didn’t want to even have a baby, actually, it is a stressful period” (IDI042022Makongoro). [SIC]*

### Practical and material support

Participants emphasized the importance of assistance with childcare and household responsibilities. In one focus group discussion, a mother explained that

“We need family support during the postpartum period so that we can have time to rest.” (0401FGD).

Another mother stated that*, “I would like someone to look after the baby so I could sleep at least for an hour” (IDI032022R1Hosp)*.

### Lived experiences of young and first-time mothers

The findings revealed several key themes regarding the postpartum lived experiences of young and first-time mothers, including positive experiences, such as pride in motherhood, confidence and responsibility, and emotional bonding with their newborns. Negative experiences were also reported, such as sleep disturbances, physical challenges (e.g., changes in appetite and weight), challenges with childbirth and breastfeeding, and emotional distress, including anxiety and depression (see Table 3).

**TABLE 3:** Lived experiences of first-time mothers during the postpartum period (Mwanza region, Tanzania,2022).

Theme	Sub-theme	Description
Positive experiences	Pride in motherhood	Feeling proud, fulfilled, and socially valued as a mother
	Emotional bonding with newborns	Emotional attachment, love, and closeness with the newborn
	Confidence and responsibility	Increased self-confidence and a sense of responsibility
Negative experiences	Lack of appetite and weight loss or gain	Appetite changes, weight fluctuations, and fatigue
	Sleep disturbances	Lack of sleep due to infant care demands
	Childbirth and breastfeeding difficulties	Difficulties due to a lack of experience or knowledge
	Emotional distress	Feelings of stress, anxiety, and depression

### Positive post-partum experiences of young and first-time mothers

#### Pride in motherhood

Participants expressed joy, fulfillment, and a sense of identity associated with motherhood. One mother shared the following during an in-depth interview.

“I am very grateful and proud to be the mother of this child.” *(IDI042022Buzuruga),*

Another mother expressed*. “I stayed for more than five years without having a baby…. but I am proud that even in my husband's family they will feel good” (FGDO42022 Makongoro).*

The desire to have a baby was evident among the study participants; for instance, one mother stated,

“*I am fully satisfied with having this child because we have increased in the family and it’s God's glory”* (*IDI 012022Buzuruga*).

Another *participant* shared that *“it feels proud to be a mother in the community” (IDI022022Nyamagana).*

#### Emotional bonding with their newborns

Strong emotional attachment and affection toward newborns were commonly reported. One of the study participants pointed out that

“Being in love with your baby to me is a feeling I am proud of” (IDI 042022Bugarika).

Another participant added, *“To show love and care is normal to a newborn baby, and sometimes I forget even if the father of the baby is present … all the time I pay attention to my newborn baby” (FGD 012022Makongoro).[SIC]*

Another mother added, *“To me, being a mother means confidently enough and getting to see all the possibilities in the world through your children’s eyes, and also wanting to be the kindliest and most generous version of yourself, so that your children can look up to you” (IDI02022R1Hosp).[SIC]*

#### Confidence and responsibility

Motherhood was associated with increased confidence and a sense of responsibility. One participant shared *that*

“Being a mother means taking responsibility to provide a happy and meaningful life for my child.” Fwith (IDI022022Makongoro).

### Negative postpartum experiences of young and first-time mothers

#### Lack of appetite and weight loss or gain

The majority of the study participants defined the lack of appetite and weight gain or loss as one of the outcomes of childbirth, which can vary from one mother to another. One participant stated,

“I feel like I have lost weight and my appetite has decreased, but I try to eat small amounts frequently so that my baby can have enough milk” (IDI042022Nyamagana). *(IDI042022Nyamagana)*.

Another woman, during a focus group discussion, shared that

“It is stressful to have gained so much weight while having a very high appetite, but what can I do? I am told it will be fine once my baby is healthy, and my weight will gradually return to normal” (FGD032022Bugarika).

#### Sleep disturbances

Sleep disturbances were commonly experienced by young and first-time mothers during the early postpartum period. One study participant described this as follows:

“To me, I have not slept well since I delivered this baby, it’s too much and tiresome you need to breastfeed and at the same time to change her clothes” (IDI022022Buzuruga).[SIC]

A young mother added*, “you had a wound from caesarian section, and the baby is crying, you will not be comfortable sleeping” (IDI042022Nyamagana)*. [SIC]

#### Childbirth and breastfeeding difficulties

Young and first-time mothers revealed childbirth and breastfeeding were difficult, as these were their first experiences and they lacked knowledge on how to care for and feed their newborns. One mother, during an in-depth interview, shared that

*“This was too hard for me as I did not have enough milk after the caesarean section and the baby was crying a lot, especially during the first three days” (IDI032022Makongoro*). [SIC]

Another young mother revealed that *“For me, some of the family members insisted I should take some herbal medication to have enough milk for the baby” (IDI032022Buzuruga).*

A first-time mother in a focus group discussion added, *“As a first-time mother, I lack a lot of skills and experiences as I have never breastfed before, classes and demonstrations are needed, unfortunately, I did not get such a chance” (FGD022022Buzuruga). [SIC]*

#### Depression and anxiety

Depression and anxiety were among the challenges faced by young and first-time mothers, who often worried about caring for their newborns and experienced hormonal changes. One participant highlighted that

*“Sometimes I worried about how to take care of my baby, and I didn’t have any experience, nor did I have enough milk, this was so stressful to me*” *(IDI032022R1Hosp). [SIC]*

Young and first-time mothers also reported issues such as a lack of informed caregivers at home, supportive relatives and families, well-trained midwives, and financial capability, and said that they would have liked to have solutions for these for their next pregnancy.

The majority of respondents mentioned the need for a knowledgeable caregiver; family and relatives will help the mother to take care of her newborn baby without worries. During an in-depth interview, one first-time mother stated that

“*First time mother and other members of the family need to be well knowledgeable on caring for the baby, to me it was so hard because I didn’t have anybody; I had to learn from the Internet” (IDI012022Bugarika)*. [SIC]

The participants also emphasized the need for an adequate number of well-trained and competent healthcare workers to help reduce hospital-based neonatal mortality. One participant, during a focus group discussion, pointed out that

“*I witnessed one of my friends, her baby died at the hospital because there was none of the healthcare workers to assist on that night shift*” [SIC] (FGD022022R1Hosp). Another participant, during an in-depth interview, added, “*It is so painful, carrying a baby for nine months and after a successful delivery, the baby died*” (IDI022022Bugarika). [SIC]

## Discussion

This study highlights the complex postpartum experiences of young and first-time mothers in northwestern Tanzania. Cultural, social, economic, gender-related, and community factors shape these postpartum experiences, while gaps in knowledge about postnatal care and breastfeeding remain evident. Consistent with previous literature, limited psychological support, information, and practical assistance further exacerbate postpartum challenges [1, 6, 8, 9]. The positive aspects of pride, bonding, and confidence align with findings that joy can buffer stress and strengthen parent-infant attachment [21]. However, negative experiences (e.g., sleep issues, breastfeeding difficulties, anxiety) underscore vulnerabilities, particularly among those who have had a cesarean section and with limited family support. These echo findings from Tanzanian and sub-Saharan African studies highlighting isolation and unmet needs [9, 22].

Young and first-time mothers in northwestern Tanzania have significant postpartum necessities, some of which remain inadequately addressed. Key needs include information, psychological and material support, shared experiences, and mentorship to help them adjust to motherhood. Similar gaps in postpartum information have been reported among young and first-time mothers in England [23]. It is important to understand that the respondents are new to the role and may not have enough experience and knowledge of many postpartum issues, as far as postnatal care is concerned. In the words of one of the respondents, “I *would have liked it if someone had talked to me before”*.

A good rapport between healthcare providers and young and first-time mothers can improve their postpartum experience. In urban Tanzania, supportive midwives who communicated openly and provided emotional support contributed to better outcomes for mothers and newborns [17]. In other settings, however, mothers have been reported to require support from their spouses and extended family regarding the knowledge and skills needed for postpartum and baby care, rather than from healthcare workers [24]. For instance, in Shenzhen, it was reported that if the mothers and grandmothers agreed on child care practices, this would facilitate the transition into motherhood and ease the stress felt by postpartum women [24]. Such differences are attributed to cultural differences among communities.

Findings from this study show that young and first-time mothers may experience stress and depression because of limited experience and confidence in caring for their babies. Similar psychosocial challenges have been reported in Germany, while other studies have linked maternal distress to having a sick newborn, an emergency cesarean delivery, and severe labor pain [25]. The differences may reflect the timing of the interviews, as the majority of mothers in our study were at least 6 weeks postpartum and had begun to experience the joys of motherhood, whereas mothers in other studies were interviewed shortly after delivery while still in hospital.

Partner relationships, socioeconomic conditions, and limited social support may contribute to anxiety and depression among young and first-time mothers. Evidence from Mwanza City, showed that poor partner relationships and lower socioeconomic status were associated with higher odds of antenatal depression, with a prevalence of 33.8%, particularly among primigravidae [26].

Given the high maternal mortality burden and the limited attention given to psychosocial health during antenatal care, integrating psychosocial screening and support into antenatal and postnatal services, together with family and community involvement, may help to identify and address these challenges early [26–28].

Alongside the needs addressed at the hospital, young and first-time mothers also require adequate assistance at home to care for themselves and their babies. Our study found that mothers needed essential physical resources and emotional support, both of which were not adequately available to all participants. While healthcare workers play an important role in educating and guiding mothers during antenatal and postnatal care, partners and family members are equally important in providing practical, emotional, and financial help, particularly as the needs of mothers and their households increase during pregnancy and after childbirth. In Tanzania’s patriarchal society, where gender roles often shape the expectations placed on men and women, poverty further limits the ability of families to meet these needs. This includes the ability of fathers to provide financial and practical support, despite their willingness to help mothers and their infants [26, 29, 30].

Findings obtained from our study identified that the majority of mothers feel proud and happy after becoming mothers. This is especially true for those who have been childless for a long time. A woman who has a child is respected in the community, and motherhood signifies great responsibility. A similar study explained that the joy and positive emotions experienced during motherhood serve as a buffer against stress and are an important component of the connection between parents and children that enhances psychological health and wellbeing [21]. Hence, positive experiences during the postpartum period should be encouraged. It is important to note that the presence of social networks, such as family and friends, is important in providing resources for the wellbeing of the mother both during pregnancy and following childbirth [30].

The fact that first-time and young mothers have sleepless nights calls for immediate attention. A study by Hijazi et al. showed that good sleeping habits have a great impact on infant growth; therefore, mothers with infants who sleep well are less anxious compared to those who sleep poorly. Moreover, this study identified that sleep quantity may be affected by several factors in the sleeping environment, such as exposure to light and noise, mattress comfort, and room temperature [31]. Results from our study identified that mothers with infants who had poor sleeping patterns were restless and experienced uncomfortable sleep. However, other participants in our study said that they had plenty of time to rest because their spouses and family provided significant support with infant care during the postpartum period. Therefore, great support from family members and spouses is needed to help young and first-time mothers feel comfortable during the postpartum period.

The present study identified that mothers who delivered their infants with a cesarean section had difficulty breastfeeding their newborns in the first few days after delivery. This is consistent with previous studies, which reported that cesarean section causes depression in mothers and results in lower milk production [32]. Additional research is needed to determine the relationship between cesarean section, stress, and milk production.

The majority of young and first-time mothers in our study gained weight during the postpartum period; this may be due to changes in sleeping patterns, physical activity levels, and changes in dietary intake. A study in urban Malaysia also reported that being less active and a high-energy diet could explain variation in weight retention 6 months postpartum [33]. A study by Tahir et al. found that women who breastfed for 6 months or longer retain approximately 0.45 kg less weight 6 months after childbirth than those who breastfed for 3 months only [34]. Therefore, breastfeeding should be encouraged because it helps control weight and strengthens the health of the infant. Based on the findings of this study, there is a need to enhance user-friendly antenatal and postnatal services for first-time and young mothers to minimize detrimental effects. For instance, there is a need to improve the relationship between mothers and midwives, which has emerged as poor. The overall relationship will improve care and safety assurance for the mother and the baby throughout the entire postpartum period. It is vital to establish strategies to ensure that mothers' needs are met and that they are helped through their negative experiences more quickly by their partners and family members, who need to be educated in their respective communities. One cannot overemphasize the need to continue educating women, encouraging them to practice exclusive breastfeeding during the entire period of 6 months, as studies have shown this to be an effective way to control weight and ensure the good health of the baby. Well-trained and equipped healthcare workers play a crucial role in ensuring that the journey of a mother during postpartum is safe. There is a need, therefore, to continue putting more effort into continuous on-the-job training and increasing the number of healthy healthcare workers.

### Strengths and limitations

Key strengths of this study include methodological triangulation, the achievement of data saturation, and capturing rich participant perspectives. Our findings are limited by reliance on self-reports (no provider views) and the exclusion of women with severe complications, potentially underrepresenting extreme experiences. Future research should incorporate longitudinal mixed-methods designs and provider perspectives.

### Conclusion

Young and first-time mothers in northwestern Tanzania experience significant postpartum needs and challenges alongside moments of joy and growth. Targeted interventions, such as enhanced information provision, strengthened social support, family engagement, and improved postnatal services, are essential to address these issues and reduce maternal/infant risks in high-burden settings.

## Data Availability

The raw data supporting the conclusions of this article consist of qualitative interview transcripts. Due to ethical and confidentiality considerations, the de-identified transcripts are available from the corresponding author upon reasonable request and subject to approval by the relevant ethics committee, in accordance with institutional and ethical guidelines.
